# Short-term outcomes of robot-assisted minimally invasive esophagectomy for esophageal cancer: a propensity score matched analysis

**DOI:** 10.1186/s13019-018-0727-4

**Published:** 2018-05-23

**Authors:** Haiqi He, Qifei Wu, Zhe Wang, Yong Zhang, Nanzheng Chen, Junke Fu, Guangjian Zhang

**Affiliations:** Department of thoracic surgery, The First Affiliated Hospital of Xi’an Jiaotong University, 277 West Yanta Road, Xi’an, Shaanxi, 710061 China

**Keywords:** Esophageal cancer, Esophagectomy, Minimally invasive esophagectomy, Robot-assisted, Video-assisted

## Abstract

**Background:**

Minimally invasive esophagectomy (MIE) was shown to be effective in reducing the morbidity and was adopted increasingly. The robot-assisted minimally invasive esophagectomy (RAMIE) remains in the initial stage of application. This study evaluated its safety and feasibility by comparing short-term outcomes of RAMIE and video-assisted minimally invasive esophagectomy (VAMIE).

**Methods:**

Between March 2016 and December 2017, 115 consecutive patients underwent RAMIE or VAMIE at our institute. The baseline characteristics, pathological data and short-term outcomes of these two group patients were collected and compared. RAMIE patients were propensity score matched with VAMIE patients for a more accurate comparison.

**Results:**

Matching based on propensity scores produced 27 patients in each group. After propensity score matching (PSM), the baseline characteristics between the two groups were comparable. The operation time in RAMIE group was significantly longer than that in VAMIE group (349 and 294 min, respectively; *P* < 0.001). The blood loss volume in RAMIE group was less than that in VAMIE group (119 and 158 ml, respectively), but with no statistically significant difference (*P* = 0.062). There was no significant difference between the two groups with respect to the mean number of dissected lymph nodes (20 and 19, respectively; *P* = 0.420), postoperative hospital stay (13.8 and 12.7 days, respectively; *P* = 0.548), the rate of overall complications (37.0 and 33.3%, respectively; *P* = 0.776) and the rates of detailed complications between the two groups.

**Conclusions:**

The short-term outcomes of RAMIE is comparable to VAMIE, demonstrating safety and feasibility of RAMIE.

## Background

Esophageal cancer is one of the most commonly diagnosed cancers around the world. At present, esophageal cancer is the sixth and ninth leading causes of cancer-related mortality among men and women, respectively [1]. In China, it is estimated that there are approximately 477,900 new esophageal cancer cases and 375,000 deaths in 2015 [2]. For esophageal cancer, surgical resection with radical lymphadenectomy remains a critical element in the multimodality management [3], and transthoracic esophagectomy is the preferred surgical procedure worldwide, which is conducive to en bloc resection of the tumor along with the mediastinal lymph nodes [4]. However, the open transthoracic approach is associated with high rates of postoperative complications due to the surgical trauma [5]. Therefore, to reduce the morbidity as a result of surgical trauma from open procedures, minimally invasive esophagectomy (MIE) was adopted. The role of MIE has been well established in the last few years [6–9]. Nevertheless, the MIE is not routinely applied worldwide for its high technical complexity and steep learning curve [10].

As an alternative, robotic surgery may provide the minimally invasive option for more surgeons and patients, because the robotic platform provides improved visualization with a magnified three-dimensional view and improved articulation of instruments with seven degrees of freedom, and thereby allows for precise manipulation and dissection. Although the robot-assisted esophagectomy was completed as early as 2003 [11, 12], it remains in the initial stage of application [13]. At present, there were few reports comparing RAMIE with VAMIE.

Thus, the aim of this study was to determine the safety and feasibility by comparing the short-term outcomes between RAMIE and VAMIE in patients with esophageal cancer.

## Methods

### Patient selection

We retrospectively reviewed the medical records of consecutive 115 patients who underwent McKeown minimally invasive esophagectomy in our institution for esophageal cancer without any previous neoadjuvant therapy from March 2016 to December 2017. Preoperatively, all patients underwent upper gastrointestinal endoscopy, contrast-enhanced computed tomography (CT) scan of the chest and upper abdomen, pulmonary function routinely, and were evaluated as resectable esophageal cancer. This study was approved by the ethics committee of Xi’an Jiaotong University. Each patient gave consent before the operation.

### Operation method

**Fig. 1:**
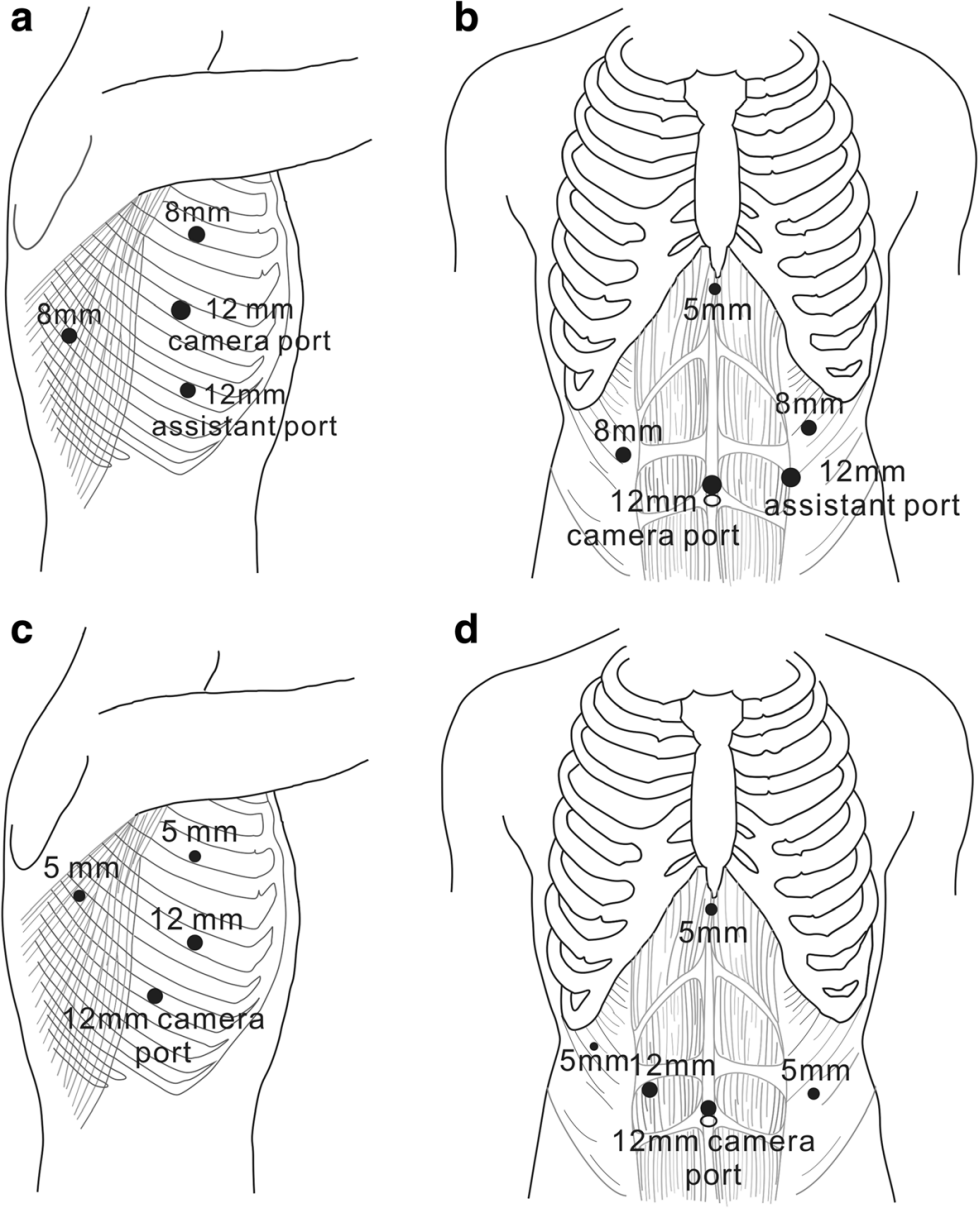
Trocar placement for thoracic part (**a**) and abdominal part (**b**) of robot-assisted minimally invasive esophagectomy, and for thoracic part (**c**) and abdominal part (**d**) of video-assisted minimally invasive esophagectomy

All patients underwent RAMIE or VAMIE with two-field lymph node dissection. RAMIE was completed using da Vinci surgical system (Intuitive Surgical, Inc., Sunnyvale, CA, USA). All patients were intubated with a double-lumen tube. During the thoracic phase, the patients were placed in the left lateral decubitus position. The trocars for thoracic part of RAMIE were placed as shown in Fig. 1a. An 8-mm robotic trocar was placed in the 3rd or 4th intercostal space (ICS) on the anterior axillary line, and another 8-mm robotic trocar was placed in the 8th ICS between the posterior axillary line to scapular line. A 12-mm camera trocar was placed in the 5th or 6th ICS on the middle axillary line. A 12-mm assistant trocar was placed in the 7th ICS on the anterior axillary line. The trocars for thoracic part of VAMIE were placed as follows: a 5-mm and a 12-mm working trocar were placed in the 4th and 6th ICS on the anterior axillary line, respectively; a 12-mm camera trocar was placed in the 7th ICS on the middle axillary line; a 5-mm assistant trocar was placed in the 6th ICS between the posterior axillary line to scapular line. Insufflation with CO_2_ at a pressure of 7–10 mmHg was used for both RAMIE and VAMIE during thoracic part. The esophagus was mobilized along with all periesophageal lymph nodes. The azygous vein was routinely ligated with Hem-o-lock clips and divided. The lymph nodes along bilateral recurrent laryngeal nerve were dissected carefully.

Trocar placement for abdominal part of RAMIE and VAMIE were shown in Fig. 1b and d. A 5-mm trocar under the xiphoid process was used in both RAMIE and VAMIE to retract the liver. When the mobilized stomach was taken out through the upper abdominal small incision, a 4-cm-wide gastric conduit was constructed with the linear tissue staplers. Then the gastric tube was pulled up to the neck through the mediastinum and a cervical end-to-side anastomosis was performed with a circular stapler.

### Data collection

The baseline characteristics, pathological data and short-term outcomes were retrospectively collected, including gender, age, body mass index (BMI), Charlson comorbidity index, forced expiratory volume in 1 s (FEV1%), tumor location, tumor grade, operation time, blood loss, the number of retrieved lymph nodes, pathological stage, postoperative hospital stay and postoperative complications. All patients were staged using the American Joint Committee on Cancer (AJCC) 7th edition TNM staging system. The postoperative complications were diagnosed and categorized based on clinical symptoms, combined with laboratory tests and radiological imaging findings. Pulmonary complications were defined as pneumonia, atelectasis requiring sputum suction bronchoscopy, acute respiratory failure, acute respiratory distress syndrome (ARDS). Recurrent laryngeal nerve injury was diagnosed at any sign of voice change or aspiration. The diagnosis of anastomotic leakage was based on definite clinical features, confirmed by esophagography and gastroscopy. Other complications were also recorded, including chylothorax, delayed gastric emptying, hemorrhage, pleural effusion and wound infection. Postoperative death was defined as death within 90 days after surgery.

### Statistical analysis

SPSS software version 22.0 was used for statistical analysis. In order to overcome the data heterogeneity, the RAMIE cases were propensity scored matched to VAMIE cases according to gender, age, BMI, Charlson comorbidity index, forced expiratory volume in 1 s (FEV1%), tumor location, pathologic T stage, and pathologic N stage. The operation time, blood loss, the number of dissected lymph nodes and postoperative complications were compared between the RAMIE and the VAMIE groups. Data were expressed as the mean ± standard deviation for continuous variables or number (%) for categorical data. Continuous variables were analyzed using Student’s test or Mann-Whitney U test, depending on normality of distribution; while categorical data were analyzed using chi-square or Fisher’s exact test. *P <* 0.05 was considered statistically significant.

## Results

A total of 115 patients with esophageal cancer met the inclusion criteria between March 2016 and December 2017. Twenty-seven patients underwent RAMIE, and 88 patients received VAMIE. The baseline characteristics were shown in Table 1. There were significant differences between the two groups in FEV1% predicted and pathologic T stage. The average pulmonary function represented by FEV1% predicted was higher in RAMIE group than VAMIE group (*P* = 0.012). Patients in the VAMIE group presented more frequently with advanced T stage (*P* = 0.042). Other characteristics were similar between the two groups.

**Table 1:** Patients’ characteristics before and after propensity score matching

Variables	Before matching			After matching		
	RAMIE (*n* = 27)	VAMIE (*n* = 88)	*P* value	RAMIE (*n* = 27)	VAMIE (n = 27)	*P* value
Gender			0.636			1.000
Male	20 (74.1)	61 (69.3)		20 (74.1)	20 (74.1)	
Female	7 (25.9)	27 (30.7)		7 (25.9)	7 (25.9)	
Age	61.0 ± 8.0	62.9 ± 8.3	0.310	61.0 ± 8.0	61.6 ± 9.8	0.621*
BMI (kg/m^2^)	21.5 ± 2.7	21.4 ± 2.7	0.935	21.5 ± 2.7	21.9 ± 2.8	0.578
FEV1% predicted	94.5 ± 13.8	84.2 ± 19.6	0.012	94.6 ± 13.8	92.9 ± 23.0	0.747
Charlson comorbidity index			0.198			0.506
1	1 (3.7)	6 (6.8)		1 (3.7)	4 (14.8)	
2	10 (37.0)	20 (22.7)		10 (37.0)	8 (29.6)	
3	13 (48.1)	37 (42.0)		13 (48.1)	11 (40.7)	
4	3 (11.1)	25 (28.4)		3 (11.1)	4 (14.8)	
Tumor location			0.457			0.514
Proximal	1 (3.7)	8 (9.1)		1 (3.7)	3 (11.1)	
Middle	18 (66.6)	48 (54.5)		18 (66.6)	15 (55.6)	
Distal/EGJ	8 (29.6)	32 (36.4)		8 (29.6)	9 (33.3)	
Histological type			0.395			0.334†
Squamous cell carcinoma	23 (85.2)	80 (90.9)		23 (85.2)	25 (92.6)	
other	4 (14.8)	8 (9.1)		4 (14.8)	2 (7.4)	
Pathologic T stage			0.042			0.334
T1	4 (14.8)	13 (14.8)		4 (14.8)	1 (3.7)	
T2	13 (48.1)	21 (23.9)		13 (48.1)	13 (48.1)	
T3	10 (37.0)	54 (61.4)		10 (37.0)	13 (48.1)	
Pathologic N stage			0.260			0.387
N0	13 (48.1)	58 (65.9)		13 (48.1)	18 (66.6)	
N1	10 (37.0)	17 (19.3)		10 (37.0)	8 (29.6)	
N2	3 (11.1)	11 (12.5)		3 (11.1)	1 (3.7)	
N3	1 (3.7)	2 (2.3)		1 (3.7)	0	
Tumor grade			0.399			0.285
Well differentiated	2 (7.4)	13 (14.8)		2 (7.4)	6 (22.2)	
Moderate differentiated	19 (70.4)	63 (71.6)		19 (70.4)	17 (63.0)	
Poorly differentiated	6 (22.2)	12 (13.6)		6 (22.2)	4 (14.8)	

Data were presented as mean ± standard deviation for continuous variables or number (%) for categorical data

*Mann-Whitney U test; †Fisher’s exact test

*RAMIE* robot-assisted minimally invasive esophagectomy, *VAMIE* video-assisted minimally invasive esophagectomy, *BMI* Body mass index, *FEV1* forced expiratory volume in 1 s, *GEJ* gastroesophageal junction

The operation time in RAMIE group was significantly longer than that in VAMIE group (349 and 294 min, respectively; *P* < 0.001). However, the blood loss in RAMIE was less than that in VAMIE group (118 and 165 ml, respectively; *P* = 0.030). The mean number of dissected lymph nodes in RAMIE group was similar to that in VAMIE group (20 and 18, respectively; *P* = 0.214). Within 90 days after surgery, there were two patients died in VAMIE group; whereas there was no patient died in RAMIE group (*P* = 1.000). There were no differences between the two groups with respect to postoperative hospital stay (13.8 and 14.1 days, respectively; *P* = 0.548) as well as the rates of overall postoperative complications (37.0 and 42.0%, respectively; *P* = 0.643) and detailed complications (Table 2).

**Table 2:** Postoperative outcomes of propensity score-unmatched and matched patients

Postoperative outcomes	Before matching			After matching		
	RAMIE (*n* = 27)	VAMIE (*n* = 88)	*P* value	RAMIE (*n* = 27)	VAMIE (*n* = 27)	*P* value
Operation time (minutes)	349 ± 45	294 ± 52	< 0.001	349 ± 45	285 ± 66	< 0.001
Blood loss (mL)	118 ± 71	165 ± 107	0.030*	119 ± 72	158 ± 82	0.062*
Number of harvested LNs	20 ± 7	18 ± 6	0.214*	20 ± 7	19 ± 5	0.420*
Postoperative hospital stay (day)	13.8 ± 2.0	14.1 ± 4.2	0.548*	13.8 ± 2.0	12.8 ± 2.7	0.128
Overall complication (at least one) [n (%)]	10 (37.0)	37(42.0)	0.643	10 (37.0)	9 (33.3)	0.776
RLN injury [n (%)]	4 (14.8)	14 (15.9)	0.891	4 (14.8)	3 (11.1)	1.000†
Pulmonary complication [n (%)]	5 (18.5)	16 (18.2)	0.968	5 (18.5)	2 (7.4)	0.224
Arrhythmia [n (%)]	1 (3.7)	8 (9.1)	0.362	1 (3.7)	0	1.000†
Anastomotic leak [n (%)]	3 (11.1)	9 (10.2)	0.763	3 (11.1)	1 (3.7)	0.351†
Chylothorax [n (%)]	0	1 (1.1)	1.000†	0	1 (3.7)	1.000†
Bleeding [n (%)]	1 (3.7)	2 (2.3)	0.556†	1 (3.7)	1 (3.7)	1.000†
Delayed gastric emptying [n (%)]	1 (3.7)	7 (8.0)	0.448	1 (3.7)	0	1.000†
90-day mortality [n (%)]	0	2 (2.3)	1.000†	0	1 (3.7)	1.000†

Data were presented as mean ± standard deviation for continuous variables or number (%) for categorical data

*Mann-Whitney U test; †Fisher’s exact test

*RAMIE* robot-assisted minimally invasive esophagectomy, *VAMIE* video-assisted minimally invasive esophagectomy, *RLN* recurrent laryngeal nerve

To reduce the bias arising from baseline characteristics such as FEV1% predicted and pathologic T stage, a 1:1 propensity score matching analysis was performed. Propensity score matching analysis produced 27 patients in each group. After PSM, patient characteristics were well balanced between the two matched groups (Table 1). The operation time was still significantly longer in RAMIE group (349 and 285 min, respectively; *P* < 0.001). Although the mean blood loss volume in RAMIE group was still less than that in VAMIE group, this difference was no longer statistically significant (119 and 158 ml, respectively; *P* = 0.062). There was no significant difference between the two groups with respect to the mean number of dissected lymph nodes (20 and 19, respectively; *P* = 0.420), postoperative hospital stay (13.8 and 12.8 days, respectively; *P* = 0.128) as well as the rates of overall postoperative complications (37.0 and 33.3%, respectively; *P* = 0.776) and detailed complications.

## Discussion

In the recent years, multiple reports have demonstrated that MIE could decrease blood loss, the length of stay, and surgical complications [6–9]. This encourages the surgeon to use and develop the minimally invasive techniques. As a novel minimally invasive technique, the robot-assisted approach has been successfully used for esophagectomy. However, the safety and feasibility of RAMIE have not been completely determined. Therefore, the present study compared the short-term outcomes of RAMIE with that of VAMIE. We found that there were no significant differences in blood loss, the number of dissected lymph nodes, postoperative hospital stay, and rates of postoperative complications, although RAMIE took longer operation time than VAMIE, mainly attributing to the docking and undocking. These results suggested that RAMIE is a safe and feasible technique, comparable to VAMIE.

Because of the high technical complexity and steep learning curve, the conventional VAMIE is not routinely applied worldwide [10]. Theoretically, the magnified three-dimensional view combined with seven degrees of freedom of the articulating surgical instruments facilitates meticulous dissection and thereby can accelerate the learning curve of RAMIE and decrease the operation time [14]. Narula et al. [15] evaluated technical enhancement of robotic and laparoscopic instrumentation in the task performance, using a computerized assessment system, and they found that the tasks were performed faster and more precisely with the robotic technology than standard laparoscopy. Further study by Chandra et al. [16] compared robotic and laparoscopic-assisted suturing performance for experts and novices. For laparoscopic novices, robotic technology significantly improves performance and accuracy. For laparoscopic experts, robotic technology significantly decreases the total instrument path length. Therefore, the robot is particularly useful for performing precise dissection in limited spaces, such as the mediastinal lymphadenectomy.

Actually, the advantages of RAMIE over VAMIE have not been well confirmed so far, even though RAMIE was completed as early as 2003 [11, 12]. During the past decade, several groups have reported their results describing the safety and feasibility of the technique [13, 17–23]. Weksler et al., [24] compared 11 patients who underwent RAMIE and 26 patients who underwent VAMIE. They found that RAMIE was equivalent to VAMIE in terms of operation time, blood loss, the number of dissected lymph nodes, postoperative complications and length of stay. Yerokun et al. [7] reached the same conclusion by comparing the short-term outcomes of 117 cases with RAMIE and 117 cases with VAMIE. In terms of postoperative complications, most previous studies [24–27] together with our present study suggested that the rates of complications were comparable between RAMIE and VAMIE, although a higher rate of anastomotic leakage was found in the RAMIE in the study by Suda et al. [28].

The lymphadenectomy is a key step in radical esophagectomy for esophageal cancer. Lymph node dissection along bilateral recurrent laryngeal nerves (RLN) has always been a challenge in MIE due to frequent recurrent laryngeal nerve injury. The robot-assisted lymphadenectomy along bilateral RLNs was demonstrated to be technically feasible and safe [29]. The study by Park et al. [25] included 62 RAMIE and 43 VAMIE. They found that RAMIE yielded more number of dissected lymph nodes than VAMIE. The same conclusion was drawn by Deng et al. [27] in a recent study, which included 42 patients in both RAMIE and VAMIE groups. Suda et al. [28] showed that RAMIE reduced the incidence of RLN injury, although it did not increase the number of harvest lymph nodes. Chao et al. [26] found that RAMIE yielded more lymph nodes along the left RLN than VAMIE. However, other previous studies [7, 24] together with our study suggested that the number of dissected lymph nodes of RAMIE was comparable to that of VAMIE. These results varied from series to series may be due to their different experience.

Anyway, this study combined with previous studies suggested that RAMIE is a safe and feasible technique, and its efficacy is comparable to VAMIE in terms of short-term outcomes. However, the limitation of our study is that it has a small sample size and it is a non-randomized controlled study. At present, there aren’t large-scale studies comparing these two minimally invasive technologies for esophagectomy. Interestingly, an ongoing randomized controlled trial by van der Sluis et al. [30] are now comparing robot-assisted with conventional open transthoracic esophagectomy. The result of ROBOT trial will provide more conclusive data.

## Conclusions

RAMIE is technically safe and feasible. The short-term outcomes of RAMIE are comparable to VAMIE. The advantages of robotic system may allow precise dissection of lymph nodes in the mediastinum and help us to decrease blood loss.

## Abbreviations

BMI: Body mass index
CT: Computed tomography
FEV1: Forced expiratory volume in 1 s
GEJ: Gastroesophageal junction
ICS: Intercostal space
MIE: Minimally invasive esophagectomy
PSM: Propensity score matching
RAMIE: Robot-assisted minimally invasive esophagectomy
RLN: Recurrent laryngeal nerve
VAMIE: Video-assisted minimally invasive esophagectomy
